# Supplementary material for: No benefit of higher protein dosing in critically ill patients: a systematic review and meta-analysis of randomized controlled trials
Source: PeerJ. 2024 May 21;12:e17433. doi: 10.7717/peerj.17433 (PMC11122048; doi:10.7717/peerj.17433)
Supplement: Supplemental Information 2 [file peerj-12-17433-s002.docx]

**Supplementary Material 2: Searching strategies**

**Pubmed 1558**

#1 Intensive Care Units [MeSH Terms] OR Critical Care [MeSH Terms] OR Critical Illness [MeSH Terms] OR icu [Title/Abstract] OR Critical Care [Title/Abstract] OR critically ill [Title/Abstract] OR Intensive Care [Title/Abstract]

#2 protein [Title/Abstract] OR proteins [Title/Abstract] OR proteins [MeSH Terms] OR amino acid [Title/Abstract] OR Amino Acids [Title/Abstract] OR Amino Acids [MeSH Terms]

#3 high [Title/Abstract] OR higher [Title/Abstract] OR low [Title/Abstract] OR lower [Title/Abstract] OR standard [Title/Abstract] OR supplemental [Title/Abstract]

#4 randomized controlled trial [MeSH Terms] OR random* [Title/Abstract]

#1 AND #2 AND #3 AND #4

**Embase 2353**

#1 ‘Intensive Care Units’:ti,ab,kw OR ‘Critical Care’:ti,ab,kw OR ' intensive care unit '/exp OR ' Intensive Care '/exp OR ‘Critical Illness’:ti,ab,kw OR ‘critically ill’:ti,ab,kw OR ‘Intensive Care’:ti,ab,kw OR ‘ICU’:ti,ab,kw

#2 ' protein ':ti,ab,kw OR ' proteins '/exp OR ' amino acid ':ti,ab,kw OR ' amino acids ':ti,ab,kw

#3 ' high ':ti,ab,kw OR ' higher ':ti,ab,kw OR ' low ':ti,ab,kw OR ' lower ':ti,ab,kw OR ' standard ':ti,ab,kw OR ' supplemental ':ti,ab,kw

#4 'randomized controlled trial'/de OR 'randomized controlled trial'/exp

#1 AND #2 AND #3 AND #4

**Scopus 3318**

#1 TITLE-ABS-KEY (Intensive Care Units) OR TITLE-ABS-KEY (Critical Care) OR TITLE-ABS-KEY (Critical Illness) OR TITLE-ABS-KEY (icu) OR TITLE-ABS-KEY (critically ill) OR TITLE-ABS-KEY (Intensive Care)

#2 TITLE-ABS-KEY (protein) OR TITLE-ABS-KEY (proteins) OR TITLE-ABS-KEY (amino acid) OR TITLE-ABS-KEY (amino acids)

#3 TITLE-ABS-KEY (high) OR TITLE-ABS-KEY (higher) OR TITLE-ABS-KEY (low) OR TITLE-ABS-KEY (lower) OR TITLE-ABS-KEY (standard) OR TITLE-ABS-KEY (supplemental)

#4 TITLE-ABS-KEY (randomized) OR TITLE-ABS-KEY (random) OR TITLE-ABS-KEY (randomised)

#1 AND #2 AND #3 AND #4

**Cochrane Library 2276**

#1 (Intensive Care Units):ti,ab,kw OR (Critical Care):ti,ab,kw OR (Critical Illness):ti,ab,kw OR (icu):ti,ab,kw OR (critically ill):ti,ab,kw OR (Intensive Care):ti,ab,kw

#2 (protein):ti,ab,kw OR (proteins):ti,ab,kw OR (amino acid):ti,ab,kw OR (amino acids):ti,ab,kw

#3 (high):ti,ab,kw OR (higher):ti,ab,kw OR (low):ti,ab,kw OR (lower):ti,ab,kw OR (standard):ti,ab,kw OR (supplemental):ti,ab,kw

#4 (randomized):ti,ab,kw OR (randomised):ti,ab,kw OR (random):ti,ab,kw

#1 AND #2 AND #3 AND #4
