# Supplementary material for: No benefit of higher protein dosing in critically ill patients: a systematic review and meta-analysis of randomized controlled trials
Source: PeerJ. 2024 May 21;12:e17433. doi: 10.7717/peerj.17433 (PMC11122048; doi:10.7717/peerj.17433)
Supplement: Supplemental Information 3 [file peerj-12-17433-s003.docx]

**Supplementary Material 3:** Publication bias assessment by funnel plot and Egger’s test, subgroup and sensitivity analyses.


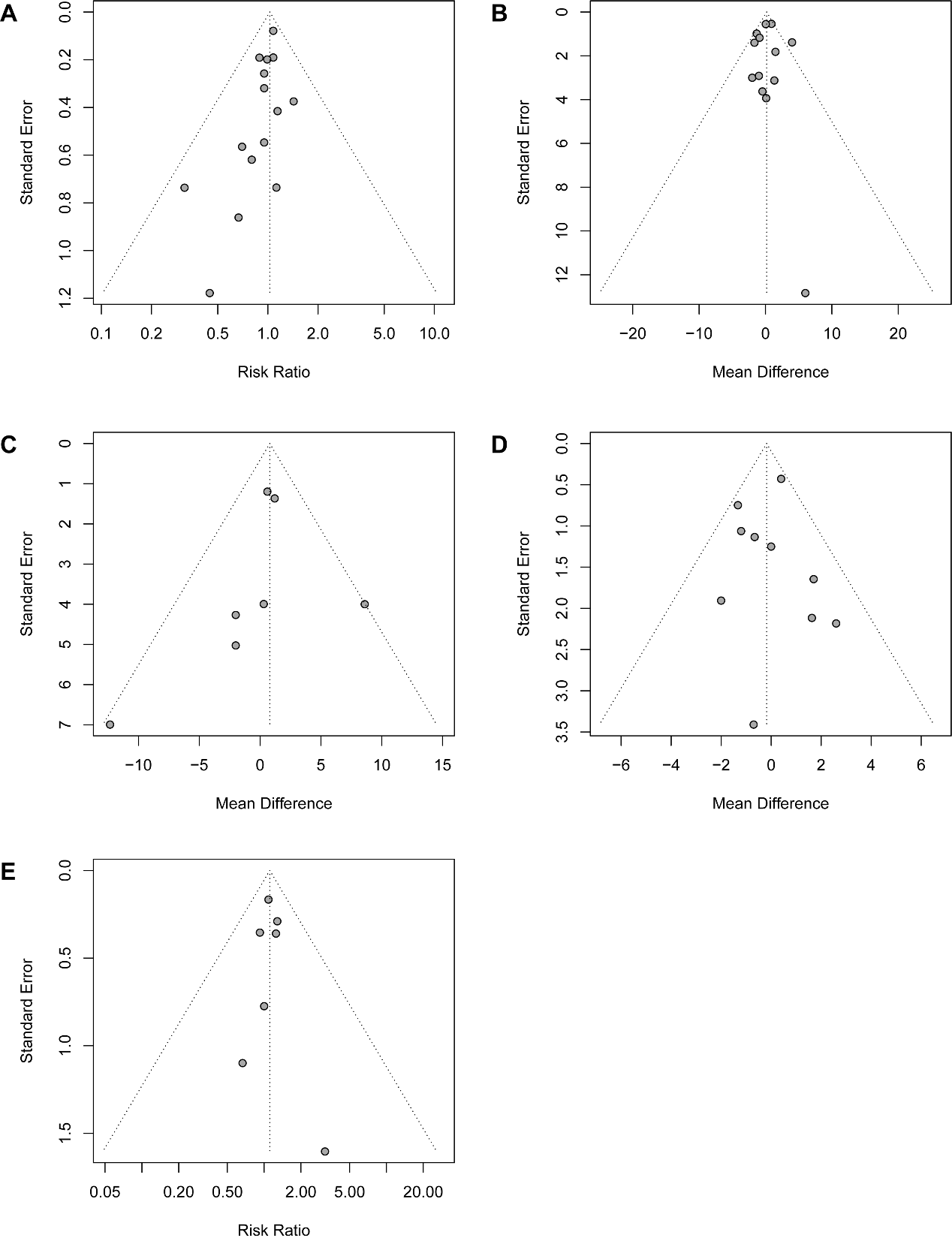


Figure S1: Funnel plot for (A) overall mortality, Egger’s test P=0.0358; (B) length of ICU stay, Egger’s test P=0.8659; (C) length of hospital stay, Egger’s test P=0.5672; (D) duration of MV, Egger’s test P=0.9480; (E) incidence of AKI, Egger’s test P=0.8192


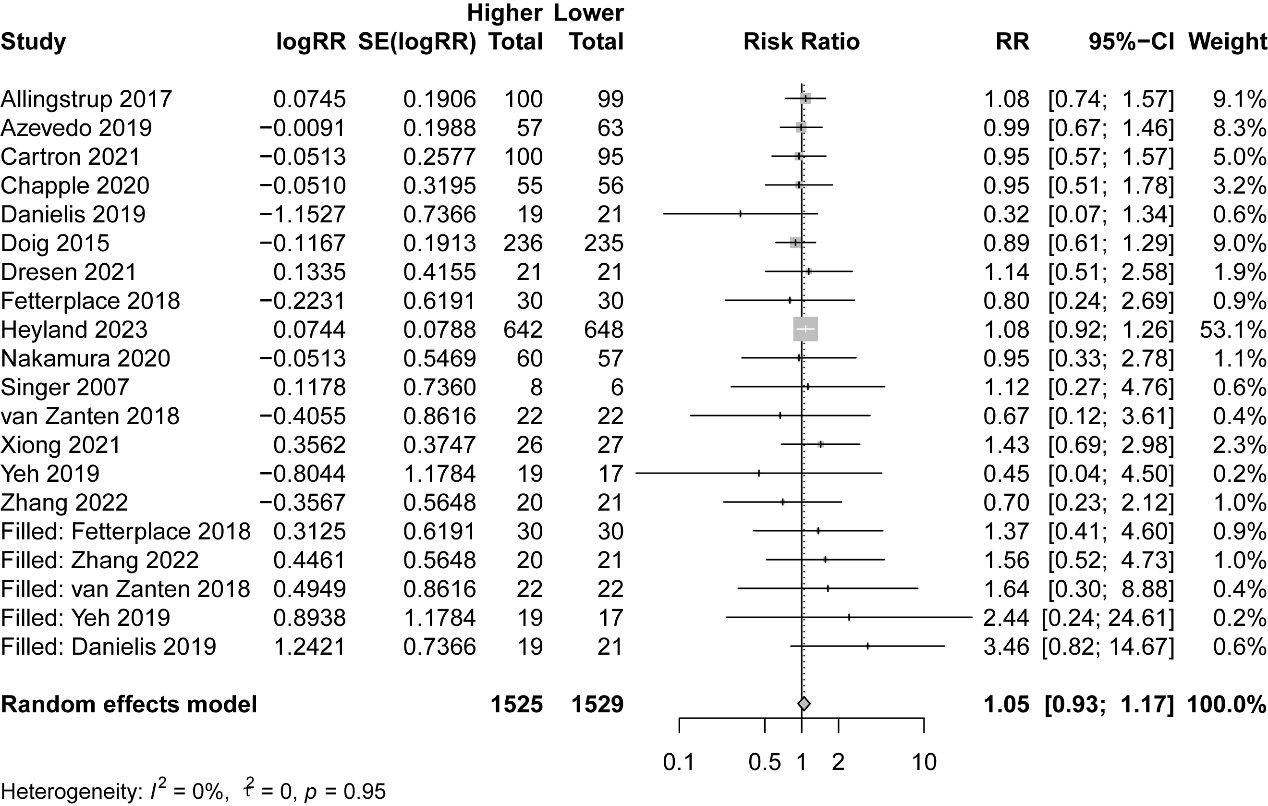


Figure S2: Forest plot after imputing for overall mortality


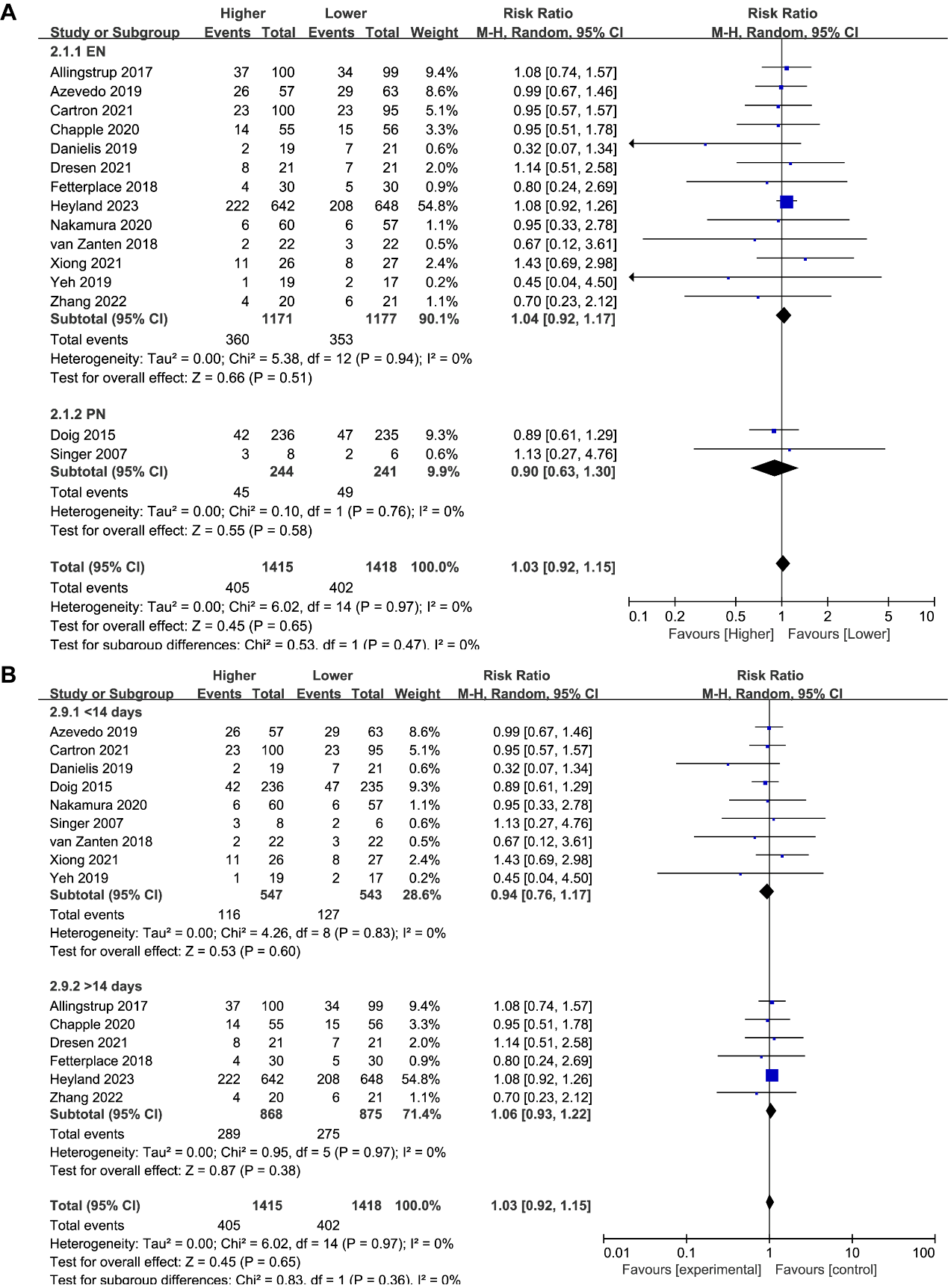


Figure S3: Subgroup analysis for overall mortality, (A) EN versus PN, (B) study duration of ≤ 14 days versus > 14 days


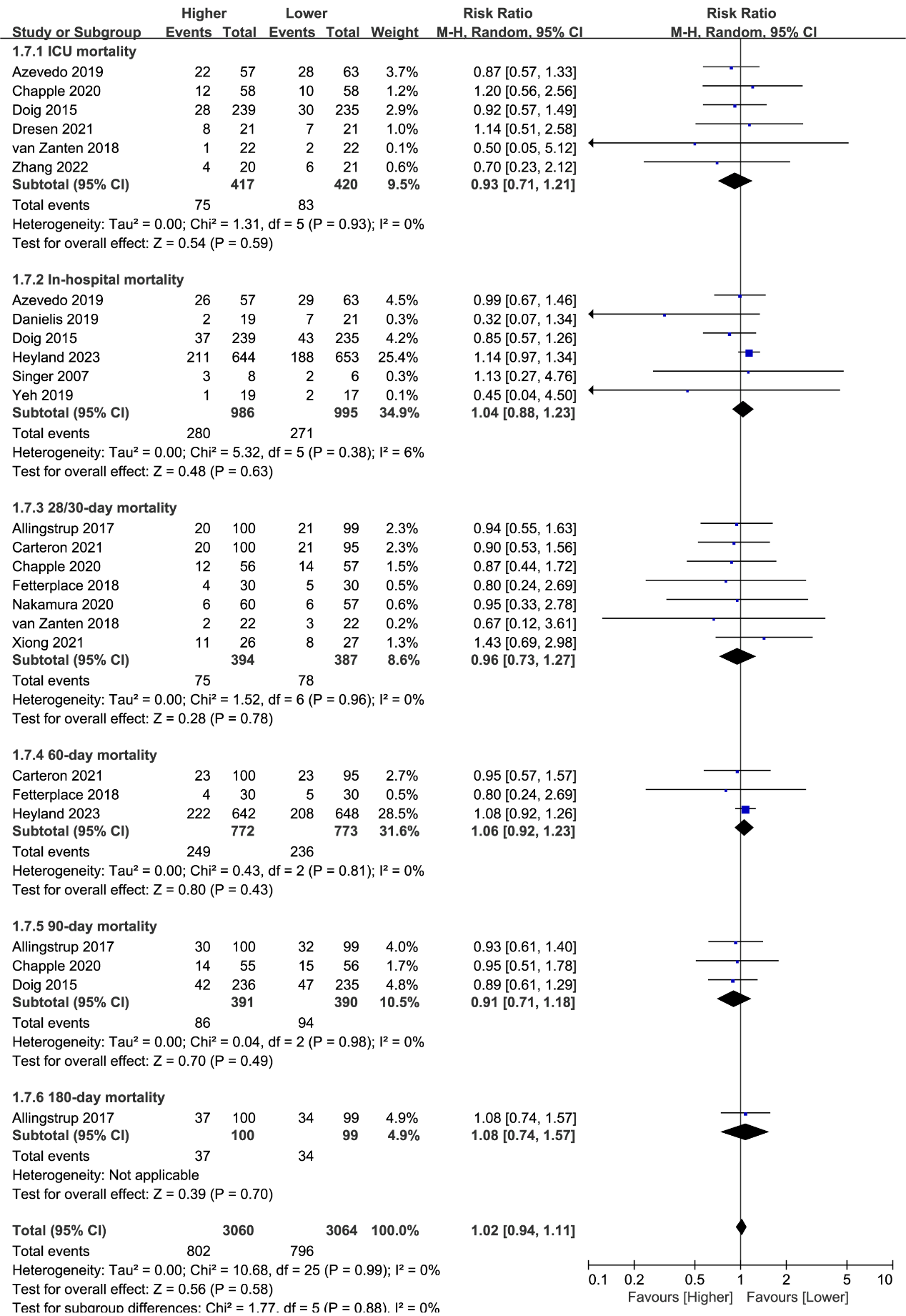


Figure S4: Subgroup analysis for overall mortality stratified by different follow-up time


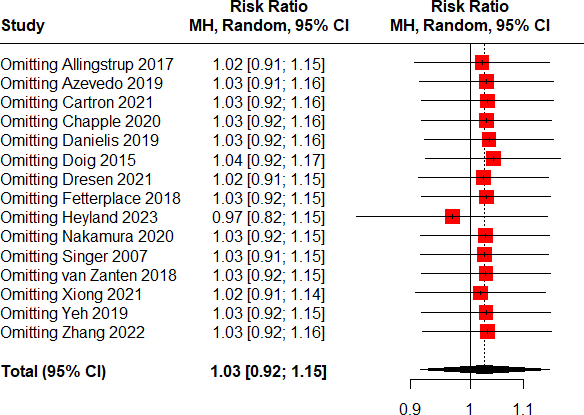


Figure S5: Sensitivity analysis for overall mortality


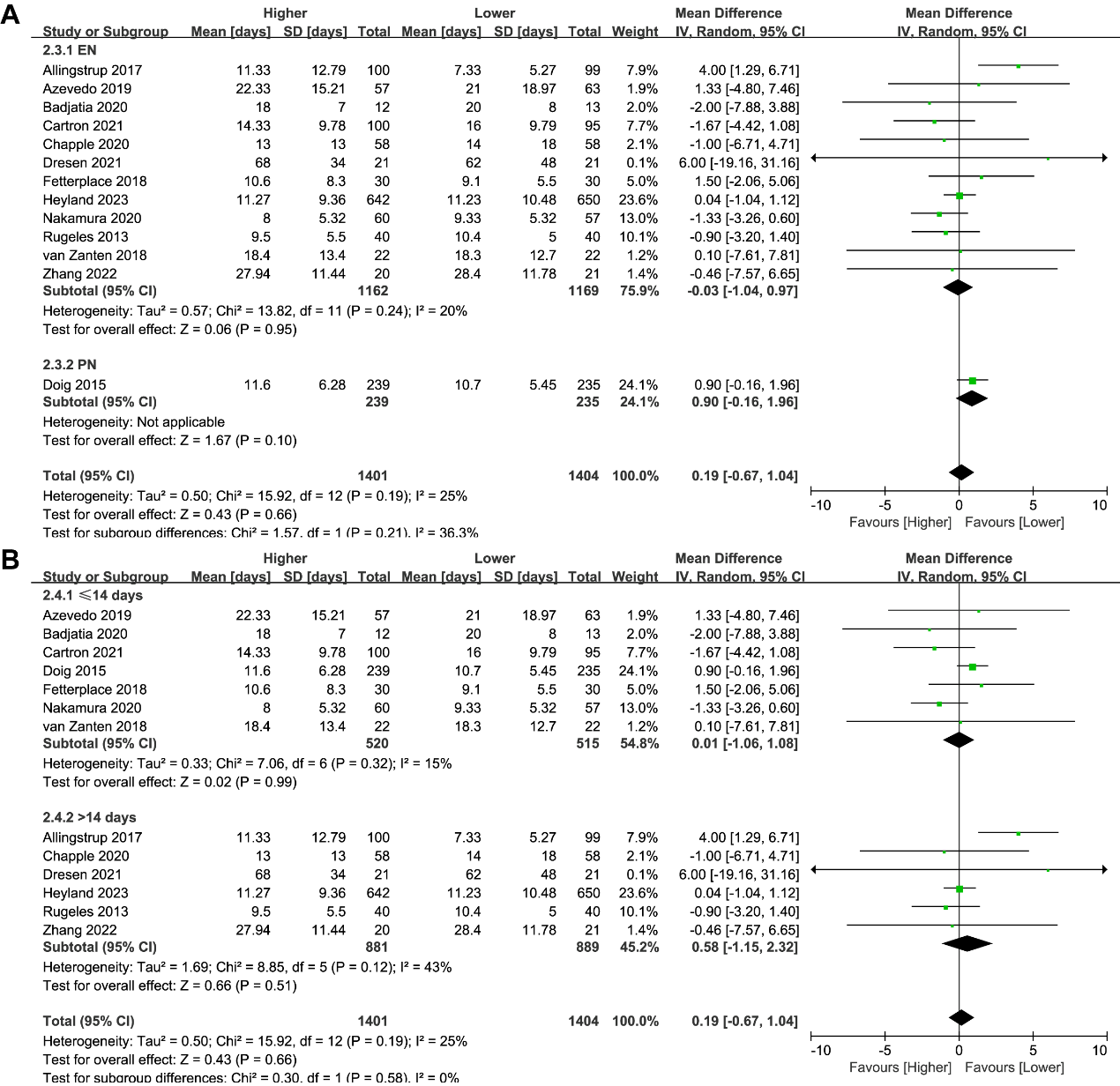


Figure S6: Subgroup analysis for length of ICU stay, (A) EN versus PN, (B) study duration of ≤ 14 days versus > 14 days


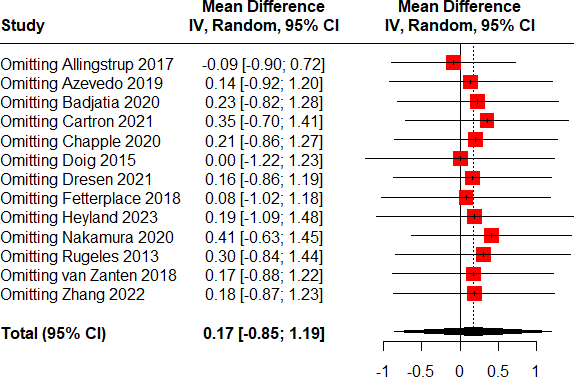


Figure S7: Sensitivity analysis for length of ICU stay


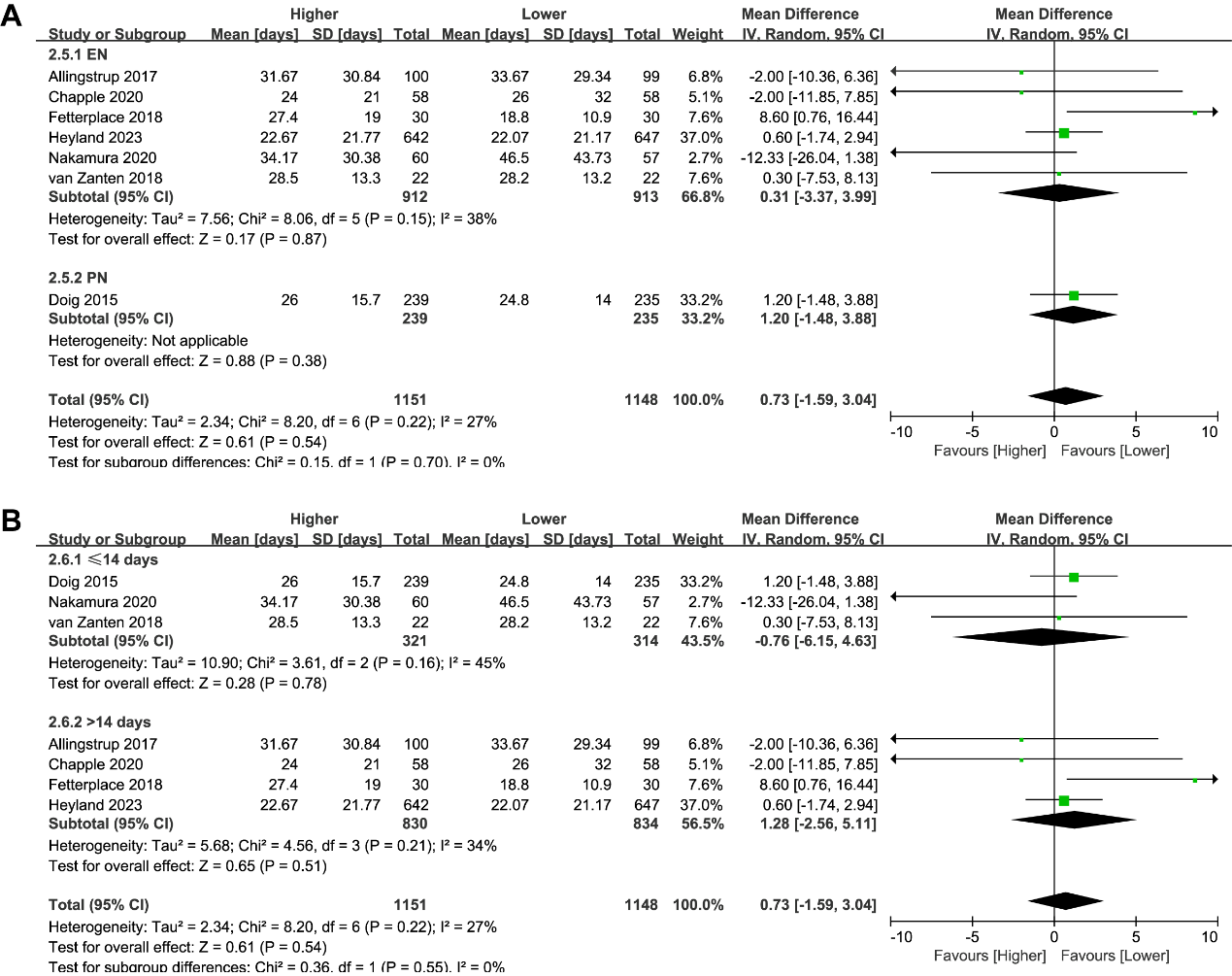


Figure S8: Subgroup analysis for length of hospital stay, (A) EN versus PN, (B) study duration of ≤ 14 days versus > 14 days


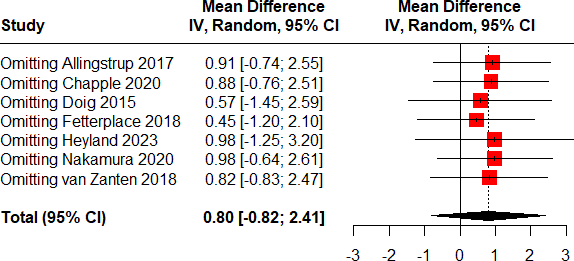


Figure S9: Sensitivity analysis for length of hospital stay


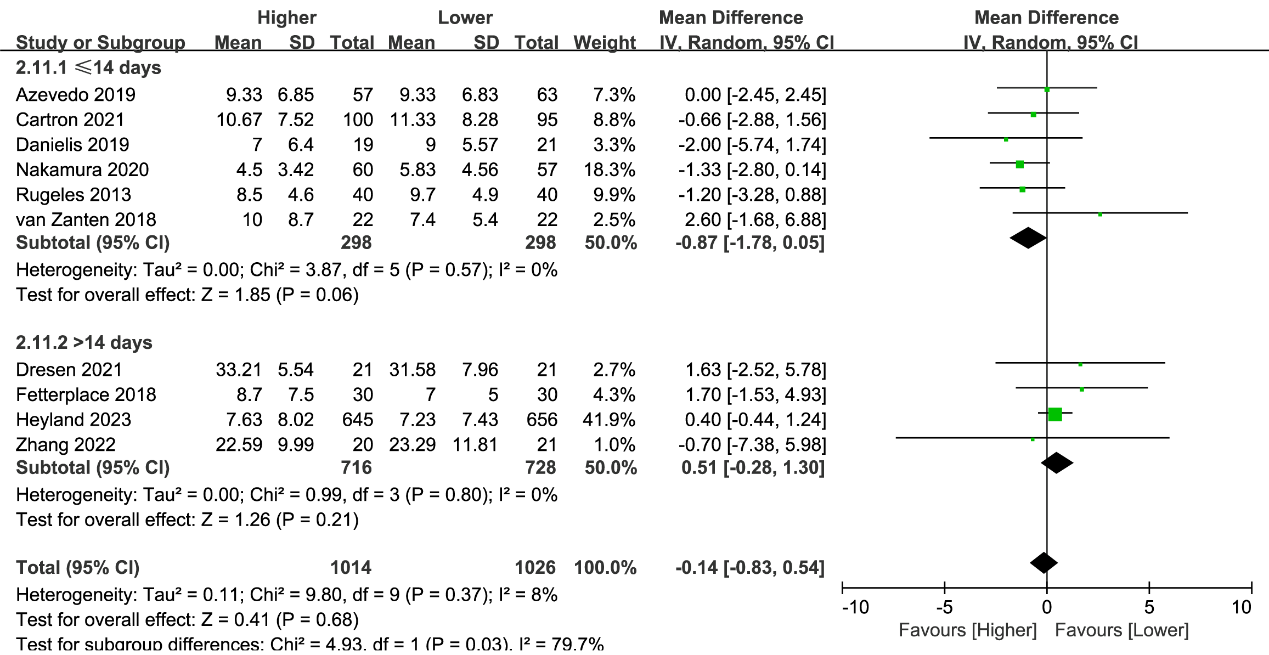


Figure S10: Subgroup analysis for duration of MV by study duration of ≤ 14 days versus > 14 days


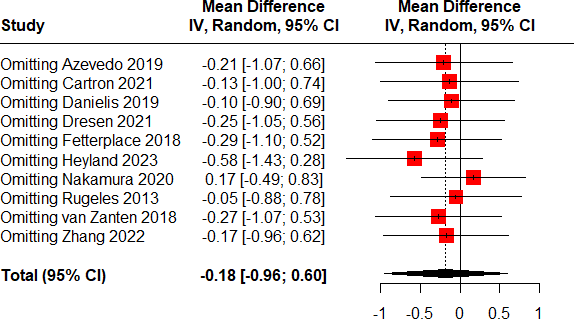


Figure S11: Sensitivity analysis for duration of mechanical ventilation


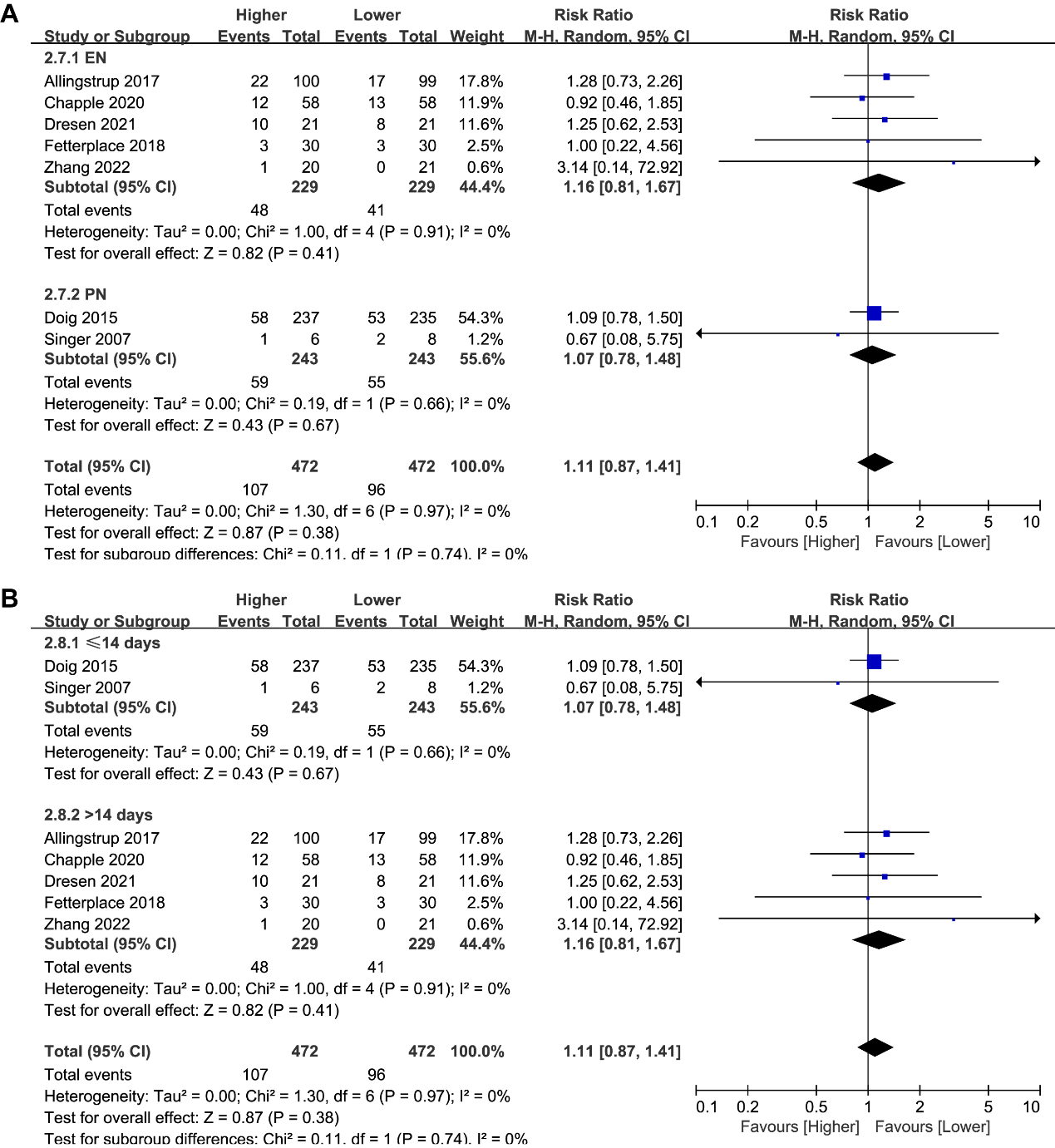


Figure S12: Subgroup analysis for incidence of AKI, (A) EN versus PN, (B) study duration of ≤ 14 days versus > 14 days


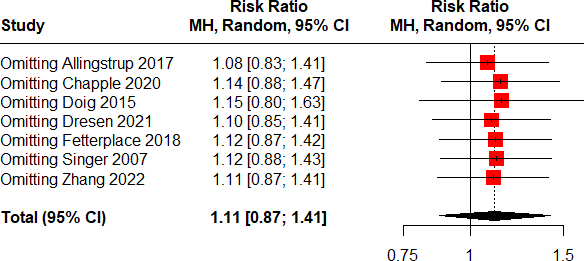


Figure S13: Sensitivity analysis for incidence of AKI


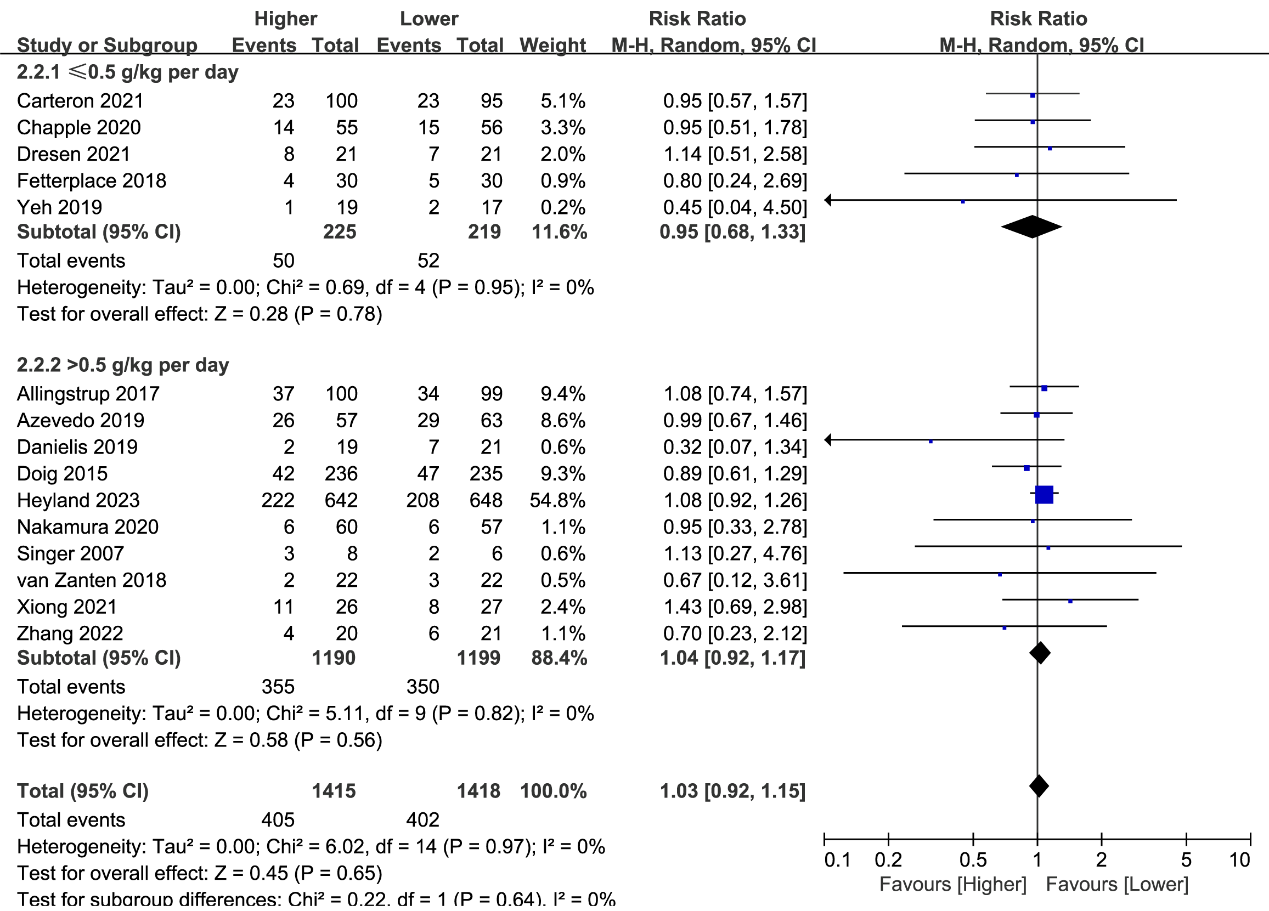


Figure S14: Post hoc subgroup analysis for mortality stratified by the difference of protein intake between higher and lower protein group (≤ 0.5 versus > 0.5 g/kg per day)


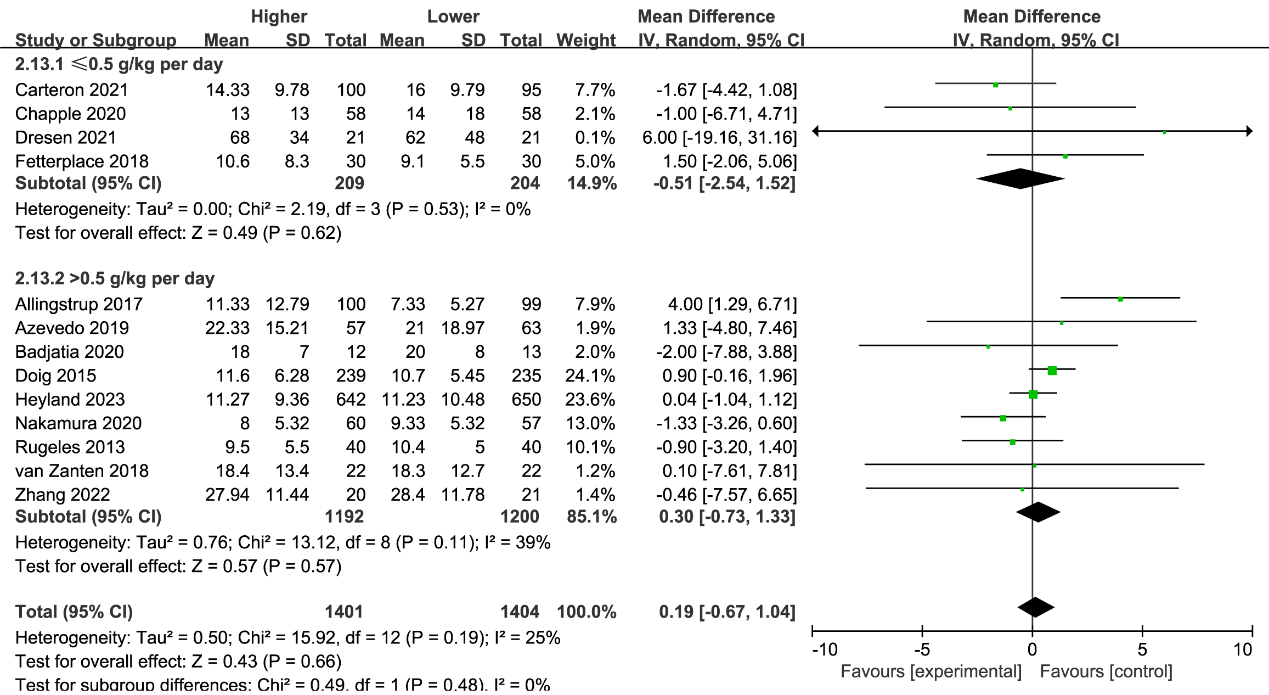


Figure S15: Post hoc subgroup analysis for length of ICU stay stratified by the difference of protein intake between higher and lower protein group (≤ 0.5 versus > 0.5 g/kg per day)


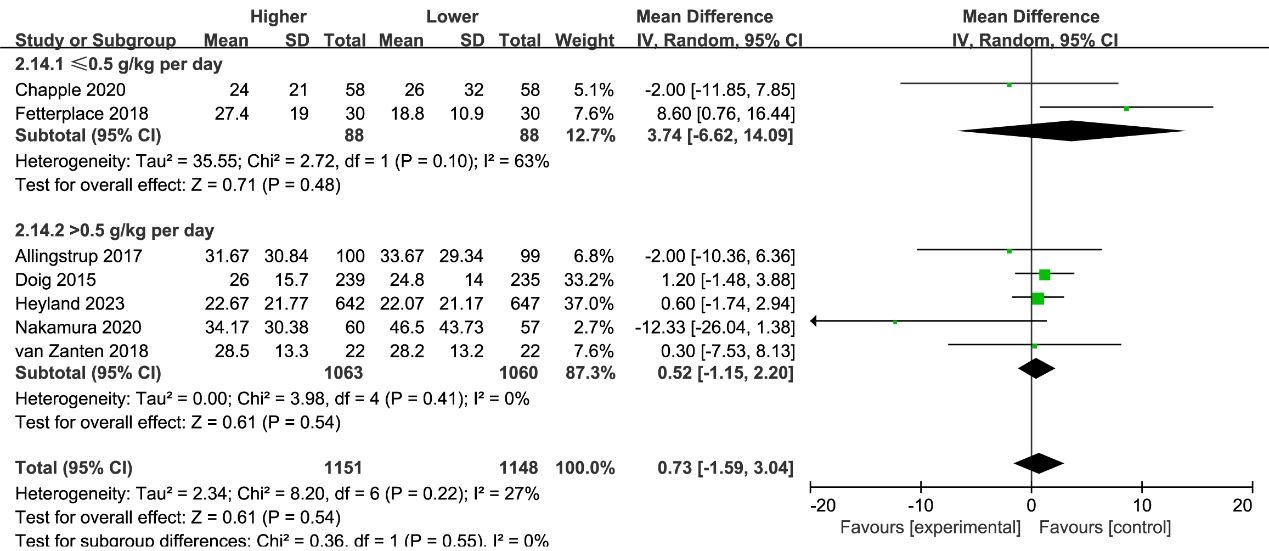


Figure S16: Post hoc subgroup analysis for length of hospital stay stratified by the difference of protein intake between higher and lower protein group (≤ 0.5 versus > 0.5 g/kg per day)


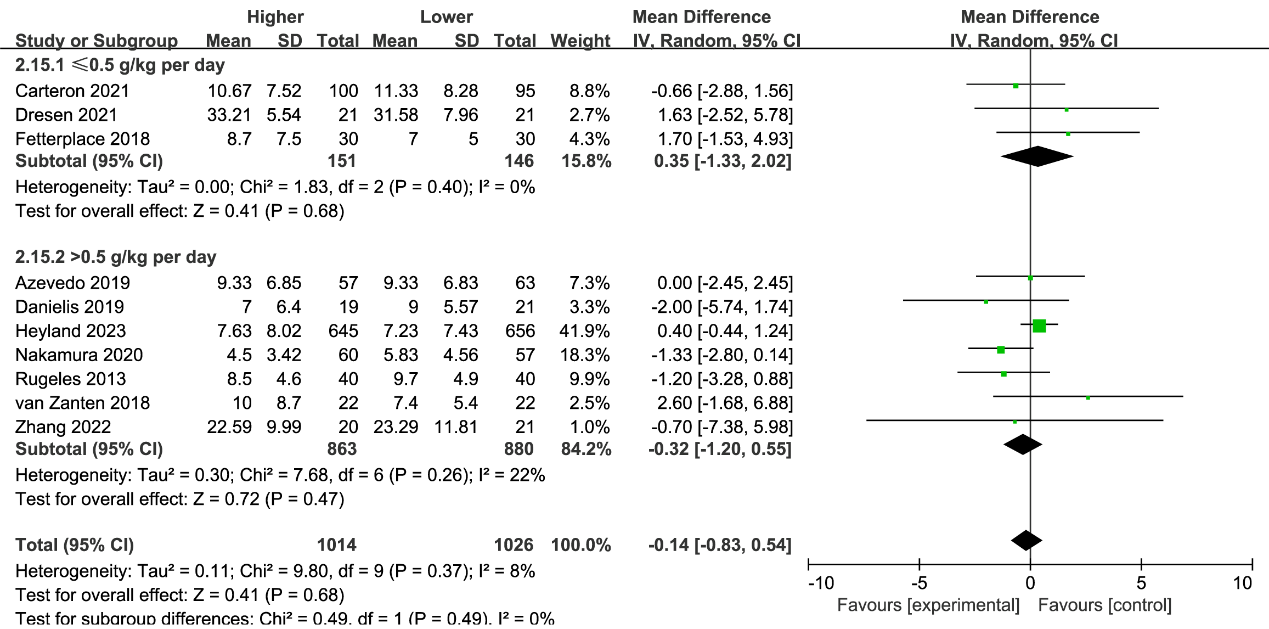


Figure S17: Post hoc subgroup analysis for duration of MV stratified by the difference of protein intake between higher and lower protein group (≤ 0.5 versus > 0.5 g/kg per day)


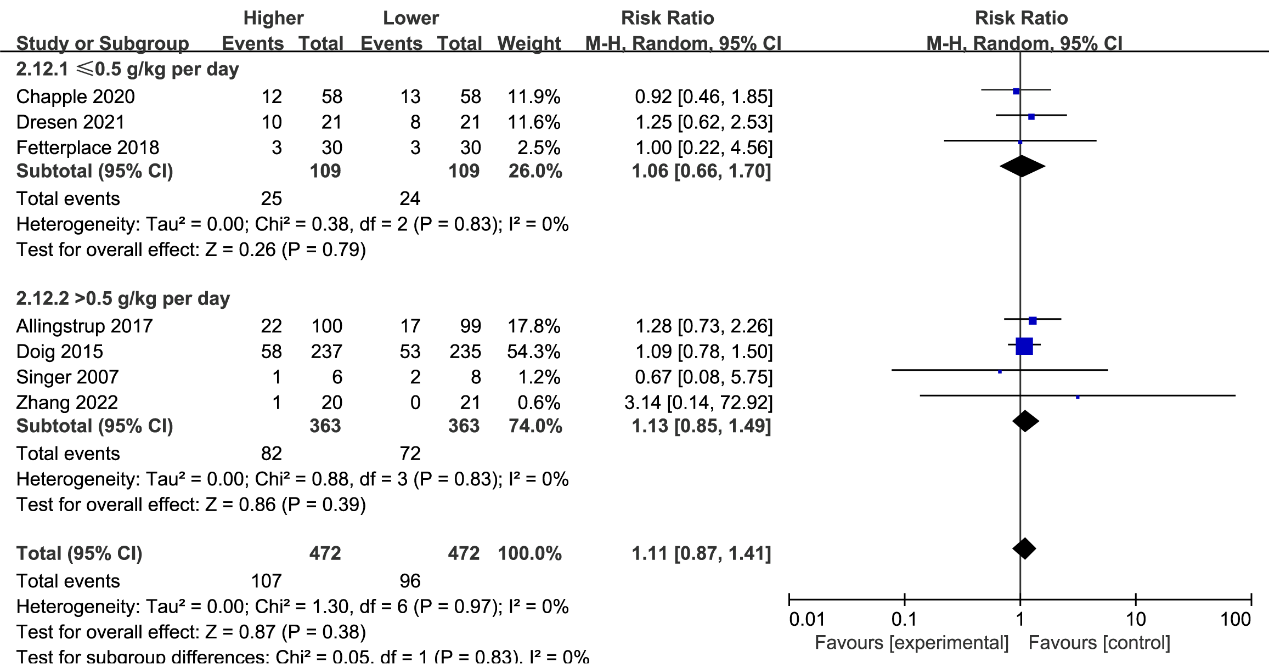


Figure S18: Post hoc subgroup analysis for incidence of AKI stratified by the difference of protein intake between higher and lower protein group (≤ 0.5 versus > 0.5 g/kg per day)
