# Supplementary material for: No benefit of higher protein dosing in critically ill patients: a systematic review and meta-analysis of randomized controlled trials
Source: PeerJ. 2024 May 21;12:e17433. doi: 10.7717/peerj.17433 (PMC11122048; doi:10.7717/peerj.17433)
Supplement: Supplemental Information 4 — The rationale for conducting the systematic review/meta-analysis. The contribution that it makes to knowledge in light of previously published related reports, including other meta-analyses and systematic reviews. [file peerj-12-17433-s004.docx]

Systematic Review and/or Meta-Analysis Rationale

1. The rationale for conducting the systematic review / meta-analysis

The optimal protein supplementation dose for critically ill patients in the ICU remains debatable. Existing RCTs investigating various protein dosage regimens have encountered limitations stemming from inadequate statistical power or inconsistent and substantial distinctions in protein dosing between groups. Limited by the weak evidence, nutrition societies worldwide recommend a diverse spectrum of protein dosages, spanning from 1.2 to 2.0 g/kg per day. Notably, specific subpopulations of critically ill patients, including those with obesity, burn injuries, or trauma, are advised to consider even more elevated protein intakes, ranging from 2.0 to 2.5 g/kg per day. Synthesizing high-quality research evidence to determine the appropriate dose of protein represents a top priority and substantial challenge for the critical care community. Consequently, we performed this meta-analysis to compare the effects of higher and lower protein doses on clinical outcomes in critically ill patients.

1. The contribution that it makes to knowledge in light of previously published related reports, including other meta-analyses and systematic reviews

Incorporating the most recent RCTs, the present meta-analysis is the most up-to-date and all-encompassing investigation of its type. Collectively, our findings align with and substantiate the outcomes documented in previous meta-analyses, thereby strengthening the existing body of evidence. Our results align well with the findings from recent RCTs, which suggest that higher protein supplementation might be harmful for critically ill patients. Patients in the high-dose protein group had higher rates of AKI, although the difference was not statistically significant.
