# Supplementary material for: No benefit of higher protein dosing in critically ill patients: a systematic review and meta-analysis of randomized controlled trials
Source: PeerJ. 2024 May 21;12:e17433. doi: 10.7717/peerj.17433 (PMC11122048; doi:10.7717/peerj.17433)
Supplement: Supplemental Information 5 [file peerj-12-17433-s005.docx]

Reasons for the addition of Xiaoya Xu as a new author:

Xiaoya Xu has played a crucial role in revising the manuscript, providing substantial input that has significantly improved the clarity, depth, and overall quality of the content. Xiaoya Xu helped to answer the comments from Reviewer 2. Since Xiaoya Xu has made substantial contributions to the revised manuscript, making Xiaoya Xu as an author both appropriate and necessary.
